# What is Diminished Virtuality? A Directional and Layer-Based Taxonomy for the Reality-Virtuality Continuum

**DOI:** 10.2196/52904

**Published:** 2024-01-31

**Authors:** Jan Egger, Christina Gsaxner, Jens Kleesiek, Behrus Puladi

**Affiliations:** 1 Institute of Computer Graphics and Vision Graz University of Technology Graz Austria; 2 Center for Virtual and Extended Reality in Medicine Essen University Hospital Essen Germany; 3 Institute for Artificial Intelligence in Medicine Essen University Hospital Essen Germany; 4 Department of Oral and Maxillofacial Surgery University Hospital RWTH Aachen Aachen Germany; 5 Institute of Medical Informatics University Hospital RWTH Aachen Aachen Germany; 6 Partner Site Essen German Cancer Consortium Essen Germany; 7 Department of Physics TU Dortmund University Dortmund Germany

**Keywords:** reality-virtuality continuum, diminished virtuality, Apple Vision Pro, VR, virtual reality, reality-virtuality, mixed reality, augmented reality, XR, extended reality, taxonomy, classification, classifications, concept, concepts, conceptual

## Abstract

The concept of reality-virtuality (RV) continuum was introduced by Paul Milgram and Fumio Kishino in 1994. It describes a spectrum that ranges from a purely physical reality (the real world) to a purely virtual reality (a completely computer-generated environment), with various degrees of mixed reality in between. This continuum is “realized” by different types of displays to encompass different levels of immersion and interaction, allowing for the classification of different types of environments and experiences. What is often overlooked in this concept is the act of diminishing real objects (or persons, animals, etc) from the reality, that is, a diminution, rather than augmenting it, that is, an augmentation. Hence, we want to propose in this contribution an update or modification of the RV continuum where the diminished reality aspect is more prominent. We hope this will help users, especially those who are new to the field, to get a better understanding of the entire extended reality (XR) topic, as well as assist in the decision-making for hardware (devices) and software or algorithms that are needed for new diminished reality applications. However, we also propose another, more sophisticated directional and layer-based taxonomy for the RV continuum that we believe goes beyond the mediated and multimediated realities. Furthermore, we initiate the question of whether the RV continuum truly ends on one side with physical reality.

## Introduction

The reality-virtuality (RV) continuum is a concept introduced by Paul Milgram and Fumio Kishino [1] in 1994. It describes a spectrum that ranges from a purely physical reality (the real world) to a purely virtual reality (VR; a completely computer-generated environment), with various degrees of mixed reality (MR) in between. This continuum is “realized” by different types of displays [2] to encompass different levels of immersion and interaction, allowing for the classification of different types of environments and experiences. The RV continuum helps us understand the varying levels of immersion and interactivity that technology can provide. As technology advances, the boundaries between these immersion levels can become more fluid, and new hybrid experiences can emerge. The continuum is particularly relevant in fields such as VR, augmented reality (AR), and MR, where researchers and developers aim to create more compelling and natural experiences that bridge the gap between the physical and virtual worlds. We used ChatGPT (OpenAI) [3] to gauge the current state of the RV continuum. According to ChatGPT, the continuum is often divided into several main categories (note, we adapted the ChatGPT results and enhanced it with concrete examples, where necessary; Textbox 1 [4]). The original ChatGPT transcript is shown in Multimedia Appendix 1 [3].

**Main categories of the reality-virtuality continuum, modified from ChatGPT.**

**ChatGPT prompt:**

- What is the reality-virtuality continuum?

**Main categories (modified ChatGPT output):**

- Physical reality (real environment): This is the state of the unmediated physical world, where all sensory perceptions are naturally experienced without any technological augmentation.
- Augmented reality (AR): In this category, virtual elements are overlaid onto the real world. AR enhances the user’s perception of the physical world by adding computer-generated visual, auditory, or haptic information. Examples include smartphone apps that display digital information on top of real-world views, such as Pokémon GO [5].
- Mixed reality (MR): MR environments combine virtual and physical elements in a way that allows them to interact in real time. Users can manipulate both virtual and real objects, and the distinction between the 2 can be blurred. Microsoft’s HoloLens is an example of an MR device that enables users to interact with holographic objects in their real-world environment [6].
- Virtual reality (VR): In VR, users are completely immersed in a computer-generated environment that can simulate various sensory experiences. VR typically involves the use of head-mounted displays and other input devices to provide a sense of presence within the virtual environment [7]. Prominent examples are the HTC Vive and the Meta Quest.
- Augmented virtuality (AV): This term is less commonly used than the others. It refers to scenarios where real-world elements are brought into a virtual environment. For example, capturing real objects or people and placing them into a virtual space. The Varjo XR-3 is capable of providing such a function and is able to make a video stream into the virtual world (VR). A concrete example could be showing a video stream of the (real) smartphone in VR, so the user can answer a text message without actually leaving VR (removing the headset and thus breaking the illusion being in “another world,” the simulated virtual environment).

## Diminished Reality

What is often overlooked in this concept is the act of *diminishing* real objects (or persons, animals, etc) from reality, rather than *augmenting* the reality with virtual things [8,9]. An introduction to the topic can be found in Cheng et al [10]. A reason for this is that diminishing something from reality needs, in general, a sophisticated understanding of the real scene or environment to make the *diminishing* aspect convincing. In AR, the real world is *just* overwritten with a virtual object. In diminished reality (DR), however, the real-world part that is *augmented* or *diminished* needs to seemingly *fit* to the reality around it. In addition, this should all be performed in real time when a user is walking around the real world, and an algorithm has to do the following (note that the first 3 items are part of the Extent of World Knowledge axis of the taxonomy by Milgram and Kishino [1]):

- Detect and track the real object that has to be removed or diminished;
- Perform geometric modeling of the scene and objects to be added or subtracted (preexisting or captured once or in real time);
- Apply the lighting model of the scene to objects added or to part of the revealed scene when something is removed (preexisting or captured once or in real time); and then
- Combine all the previous points together as the scene description for the rendering algorithm.

All of this has to be done not only in real time but also with very high precision. The inserted virtual object has to fit seamlessly into and make sense with the reality; minor discrepancies will appear to be a glitch and will be noticed immediately by the user, as we recently observed in a DR user study [11]. In fact, we think that diminution and augmentation require fundamentally different technologies. In our opinion, an augmentation may be needed to alter reality at a certain position with regard to other (real) objects (eg, displaying a patient’s tumor as an AR hologram on the patient in front of you, at the real position, such as for needle guidance [12]), but no seamless and semantic fitting is necessary. As soon as a virtual object needs to fit into the scene semantically, we consider this to require diminution. Hence, for augmentation, you *only* need a volume rendering process with some basic options, such as position, size, and transparency. For diminution, however, additional fundamentally different technologies are needed. The scene has to be analyzed and *understood*, and a meaningful replacement has to be generated and *inserted* as an AR hologram. An example could be glasses that are *removed* from a person in front of you.

In summary, the user has to get the impression that the real, diminished object does not exist at all in reality [13]. Besides sophisticated algorithms, this course of action needs a considerable amount of computing power. Fortunately, there has been tremendous progress in both areas during the last years, with deep learning–based approaches and GPUs that can run these kinds of algorithms, even in real time. As a result, DR has already found its way into some applications [5], such as virtual furniture removal for redecorating purposes (eg, IKEA Kreativ [14]). Other possible applications for DR include the following:

- Privacy enhancing: In a live video feed, certain objects or information can be blurred or removed in real time to protect sensitive or private data.
- Training and education: DR can be used to remove distractions in a learning environment or highlight specific items to focus on.
- Therapeutic applications: For someone with a phobia of spiders, a DR system could recognize spiders in the person’s field of view and diminish or replace them with less threatening images to reduce anxiety. Additionally, sensory overload, a feature of autism, could be diminished with a DR system, to reduce overstimulation.

## Directional and Layer-Based Taxonomy

**Figure 1:**
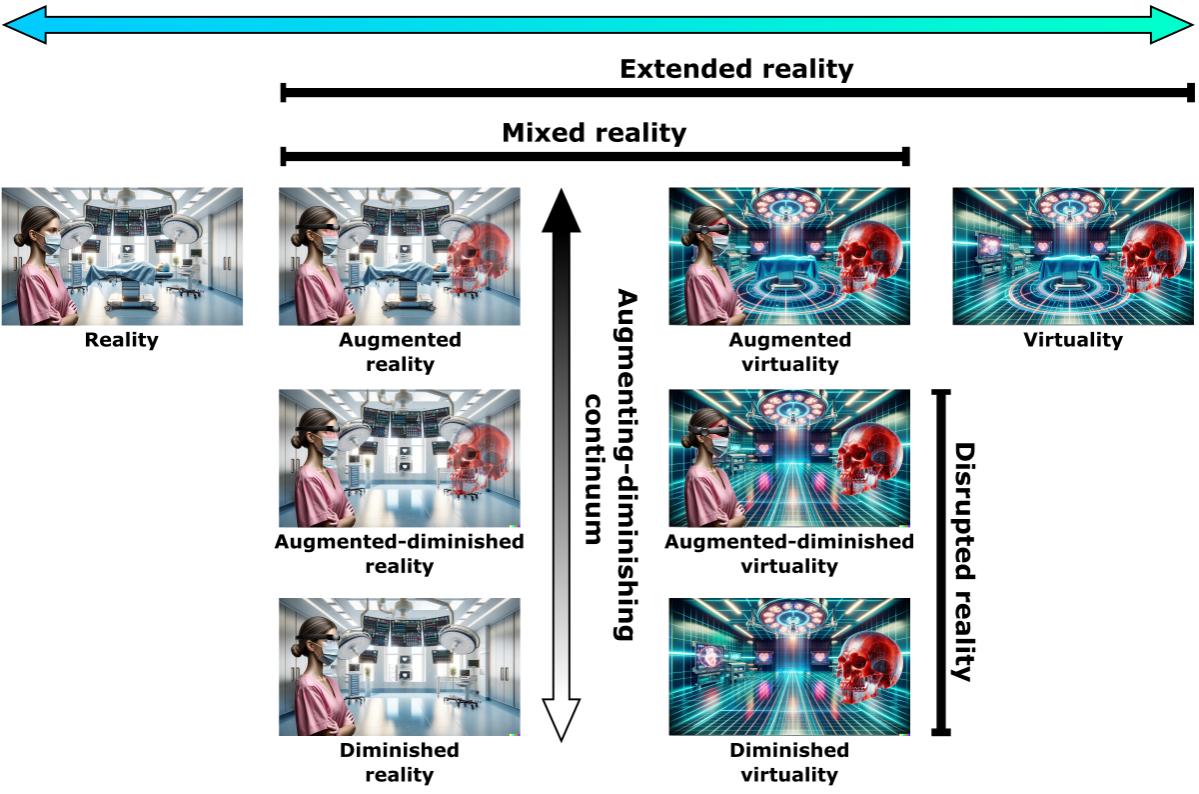
An updated reality-virtuality continuum where “diminishing” is more prominent to ensure a better understanding. The figure shows a real operation room (OR), which is “real” in the two left columns and computer-generated in the two right columns. In “reality,” a real physician is standing inside an OR without any computer-generated objects. In “augmented reality,” the real physician wears extended reality (XR) glasses in the OR and looks at a computer-generated skull of the patient to be treated. In “augmented-diminished reality,” the real OR table has been removed. In “diminished reality,” the OR table has been removed (but also note that the computer-generated skull is not visualized). On the right side is “virtuality,” that is, virtual reality (VR), where a computer-generated OR with a table and a skull are shown in VR (to a user wearing VR glasses). In “augmented virtuality,” the real physician is shown inside the VR OR. In “augmented-diminished virtuality” (ie, mediated virtuality), the computer-generated OR table is removed, but note that the real physician is still shown. In “diminished virtuality,” the OR table has been removed, but the real physician is also not shown. The “augmenting-diminishing” continuum shows the degree of augmentation and diminution (note that this does not apply for “reality” and “virtuality”). Scenarios where a diminution happens belong to “disrupted reality.” The images within the figure were generated by DALL·E 3 (OpenAI) [15] and then further modified by the authors.

**Figure 2:**
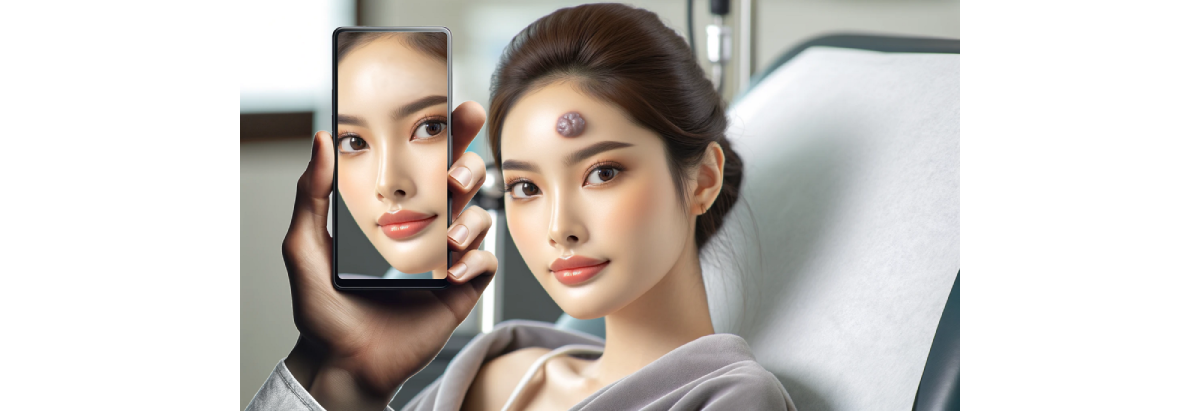
An example of the photo-editing capabilities of newer cell phones as a diminution operation, enabling the “removal” of a skin tumor virtually from a patient’s face before surgery. This figure was generated by DALL·E 3 (OpenAI) [15] and then further modified by the authors.

Nevertheless, for all these aforementioned reasons, we think that DR needs to be more prominent on the RV continuum, as shown in Figure 1 [15], without delving deeper into the broad topics of mediated reality [9] or even multimediated reality [16]. This will not only assist in the decision-making for hardware (devices) and software that are needed for new DR applications but also help unfamiliar users to get a better understanding of the entire extended reality (XR) topic (note that we are addressing this revision to the continuum purely from an application or user point of view [POV], not from the POV of an MR researcher or engineer). An example application for DR could be the real-time anonymization of a face via XR. There is a huge difference if a device detects the eye area and *simply* inpaints a black bar over the eyes (without considering the surrounding facial area) or inpaints the eyes with *different* or *meaningful* ones that fit perfectly to the surrounding facial area. The black bar approach can probably be performed on a current smartphone, whereas the second approach needs much more sophisticated hardware and computing power, with an integrated GPU that can run a trained, deep inpainting neural network in real time (note that a user with an XR headset would move around in general, which also changes the POV on the face to be anonymized, so the inpainting algorithms also has to be executed continuously in real time). In this context, we also think that the upcoming Apple Vision Pro will push the limits in DR, because it is a video-see-through device that can enable DR to reach its full potential [17]. In fact, the *Digital Crown* hardware of the Apple Vision Pro, which also exists for the Apple Watch, should enable us to seamlessly *walk* along the whole RV continuum (back and forth) and bring medical DR applications to reality, which are still almost nonexistent currently [18]. A potential example of the photo-editing capabilities of newer cell phones as a diminution operation is shown in Figure 2 [15]. In this medical example, DR enables the *removal* of a skin tumor virtually from a patient’s face before surgery.

Diminished virtuality (DV), however, remains a curiosity, and not even ChatGPT—which has been fed with 300 billion words from books, web texts, Wikipedia, articles, etc [4]—can handle this term as of January 2024 (Textbox 2; the original ChatGPT transcript is shown in Multimedia Appendix 2 [3]).

**Asking ChatGPT about “Diminished Virtuality” (as of January 2024).**

**ChatGPT prompt:**

- What is “Diminished Virtuality”?

**ChatGPT output:**

- “Diminished Virtuality” is not a widely recognized term or concept as of my last knowledge update in January 2022. It’s possible that this term has been coined or introduced in a specific context or field after that date, or it may be a term used in a niche or specialized area that is not widely known.
- If you have more context or information about where you came across this term or its usage, I would be happy to try to provide more information or insight based on that context.

**Figure 3:**
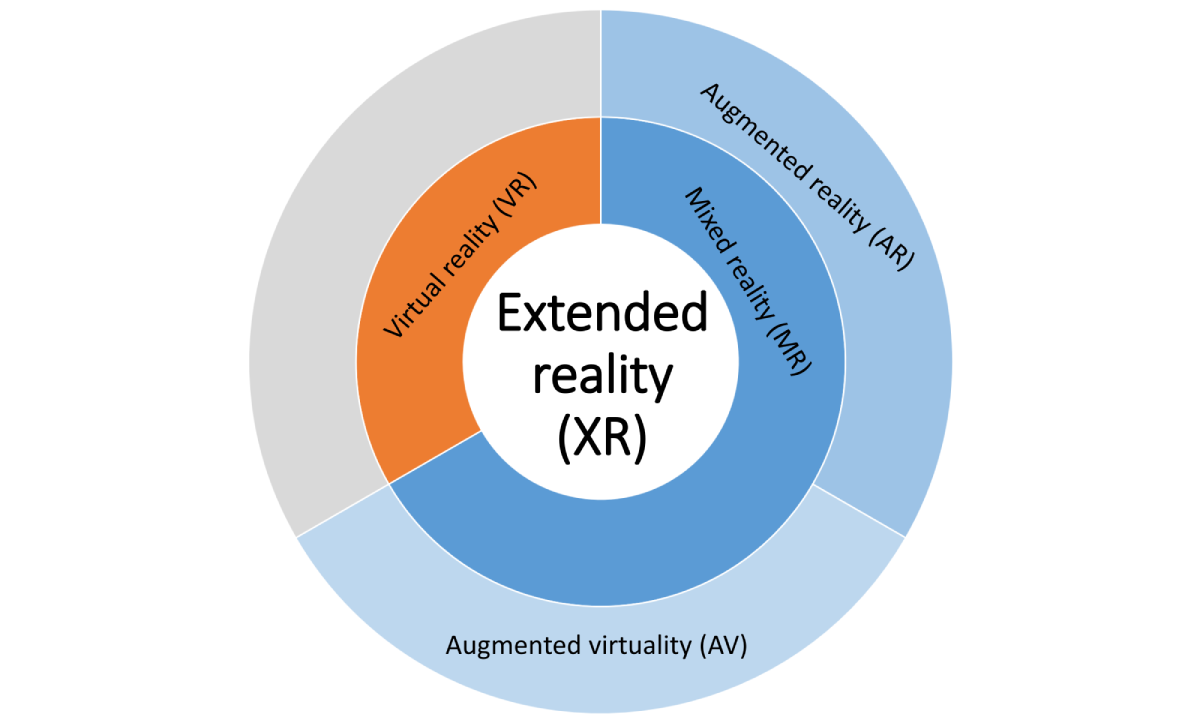
The extended reality (XR) continuum with its subsections (virtual reality [VR], mixed reality [MR], augmented reality [AR], and augmented virtuality [AV]) folded in an outside-in fashion with a circular representation.

DV can perhaps be best imagined as starting on the extreme right side on the RV continuum, which contains only virtual objects (virtual environment and VR), and going along the continuum toward the left to reach diminished virtual elements (see the figure of the RV continuum by Iriqat and Vatansever [19], and note that this is especially of interest for computer-generated structures that are very complex and where only some parts need to be diminished). This should be possible with the *Digital Crown* hardware of the Apple Vision Pro: when fully immersed, the virtuality is reduced to make the real world partly visible. Apple describes scenarios where people might want to increase the pass-through when they intend to interact with a nearby physical object or read text on another device. Another real-world scenario could be a so-called tumor board meeting that is held in VR, where several experts from around the world gather to discuss a rare case and certain virtual elements (images, texts, etc) are diminished because of privacy issues (note that the mixture of reality here is also modifiable): an externally tuned-in expert (who is traveling) can see certain patient information because he or she belongs to the clinic where the patient is located, whereas another externally tuned-in expert who does not belong to the clinic of the patient should not see or hear certain patient information. However, the full potential of DV would unfold when the virtuality is diminished in a way that also fits with the upcoming reality. An example would be a real-world person showing up in VR between virtual objects. Imagine layers of virtual and real context stacked up but still seamlessly interplay with each other for the final output. In fact, this layer-based concept could also extend to mediated and even multimediated realities, where, for example, different sounds (real and virtual) interplay with each other, depending on their location and *depth*. “Enaudio” (the “hearing” equivalent to envision) hearing real rain outside in the real world that are mixed with virtual sounds while inside a virtual room. Real and virtual input from different senses could also be mixed; for example, there is a (virtual) hole in the VR room and the (real) rain falls inside this virtual room, generating simulated sounds as they hit virtual objects in the VR room. The same also works for outputs and their combinations; for example, the real voice from someone wearing the headset is mixed with virtual sounds, or real smells are mixed into VR by generating the corresponding virtual objects there. Finally, some could argue whether the RV continuum really ends on *one side* with physical reality [20], because the brain creates reality—comparable to a head-mounted display creating VR—but does not detect it. This could be discussed and explored by the community in the future, and in this regard, we want to propose a diagram of XR that loops back (as opposed to a continuum on a straight line with 2 ends) as an inspiration (Figure 3). In summary, XR is a niche yet growing topic, because more and more consumer devices with better hardware have become available during the last few years. In parallel, artificial intelligence (AI)–based algorithms have made 1 breakthrough after another, which can, for example, be explored to develop novel applications for an intelligent health care metaverse [21].

## Appendix

Multimedia Appendix 1 Asking ChatGPT about "the reality-virtuality continuum."

Multimedia Appendix 2 Asking ChatGPT about “diminished virtuality.”

## Abbreviations

AI: artificial intelligence
AR: augmented reality
AV: augmented virtuality
DR: diminished reality
DV: diminished virtuality
MR: mixed reality
POV: point of view
RV: reality-virtuality
VR: virtual reality
XR: extended reality
