# Supplementary figures and images for: What is Diminished Virtuality? A Directional and Layer-Based Taxonomy for the Reality-Virtuality Continuum
Source: JMIR XR Spat Comput. 2024 Jan 31;1:e52904. doi: 10.2196/52904 (PMC12671302; doi:10.2196/52904)

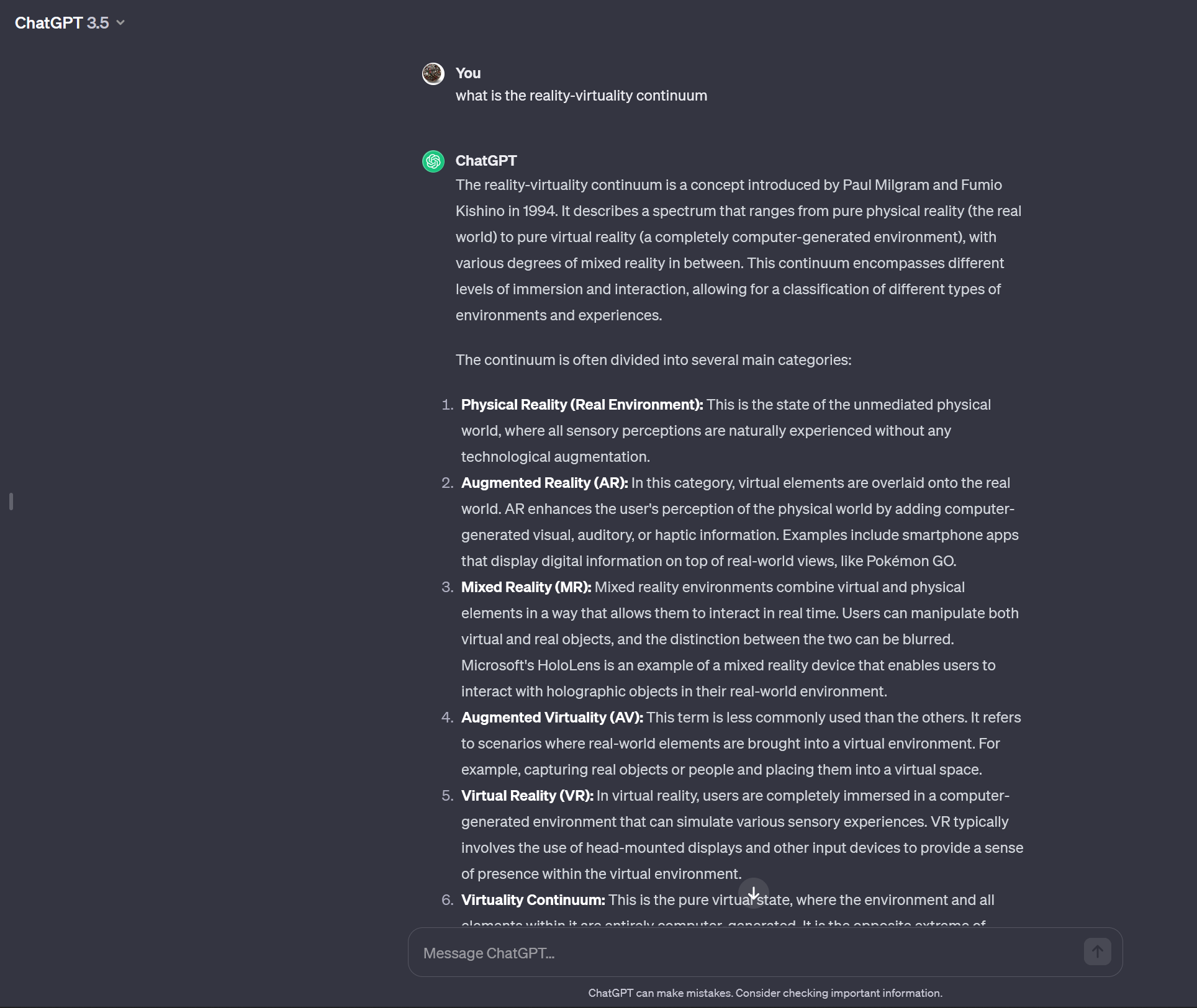

Supplement: Multimedia Appendix 1 [file xr_v1i1e52904_app1.png]

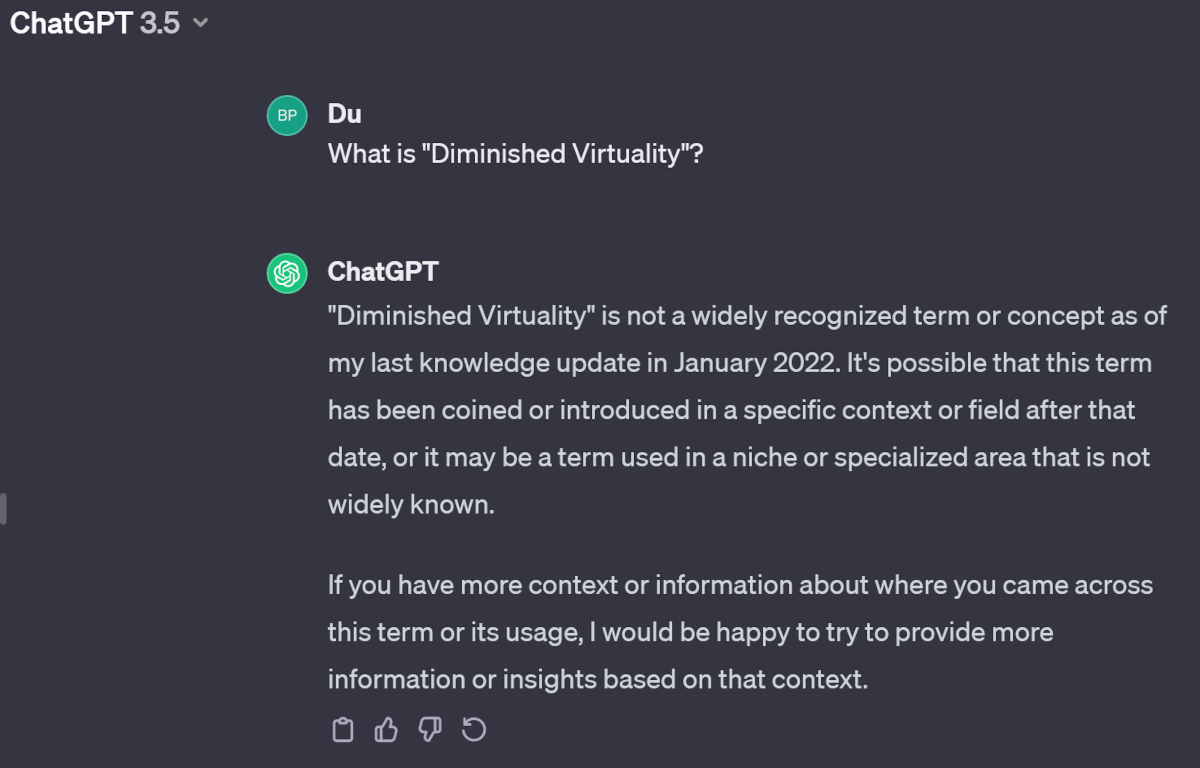

Supplement: Multimedia Appendix 2 [file xr_v1i1e52904_app2.png]
